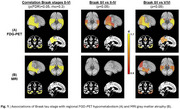# FDG‐PET Outperforms MRI in Monitoring Early Neurodegenerative Changes Tied to Neurofibrillary Tangle Pathology: An Imaging‐Pathology Study

**DOI:** 10.1002/alz70856_106413

**Published:** 2026-01-07

**Authors:** Orfeas Vourkas, Linda Zhang, Alberto Rabano, Pascual Sanchez‐Juan, Jesús Silva‐Rodríguez, Michel J. Grothe

**Affiliations:** ^1^ CIEN Foundation, Reina Sofia Alzheimer Center, ISCIII, Madrid, Madrid, Spain

## Abstract

**Background:**

Neurofibrillary tangle pathology (NFTp) in Alzheimer's disease (AD) is closely related to neurodegeneration, which can be measured in vivo using structural MRI or FDG‐PET. While several studies have assessed this correlation using in vivo markers of NFTp (i.e., tau PET), very few studies to date have compared MRI and FDG‐PET with respect to neuropathological assessments of NFTp. Here, we aimed to assess the relative sensitivities of FDG‐PET‐measured hypometabolism and MRI‐measured gray matter (GM) atrophy, to early and advanced stages of NFTp, as assessed by post‐mortem neuropathological examination.

**Method:**

We studied 88 individuals from the Alzheimer's Disease Neuroimaging Initiative (ADNI) autopsy cohort who had Braak NFTp staging performed at autopsy and had FDG‐PET and structural T1‐MRI scans acquired before death (imaging‐to‐death interval: 3.4±2.3 years). Associations of Braak stages with regional FDG‐PET SUVRs and GM volumes on MRI were assessed in exploratory brain‐wide Spearman correlation analyses across 52 cortical and subcortical brain regions. For regions showing a significant association (*p* <0.05, FDR‐corrected), we then performed pair‐wise comparisons between grouped Braak stages 0/I (*N* = 18), II‐IV (*N* = 14), and V‐VI (*N* = 56).

**Result:**

Higher Braak stages were significantly associated with lower FDG‐PET SUVR in several tempero‐parietal cortical regions typically affected by AD. Compared to the Braak 0/I reference group, most of these regions showed significant and pronounced (Cohen's d>0.9) hypometabolism in early Braak stages II‐IV, and severity of hypometabolism further increased in Braak stages V/VI (Figure 1A). Although GM volume on MRI showed a similar regional association with Braak stages, effect sizes were considerably lower and differences to the Braak 0/I reference group were only significant for advanced Braak stages V/VI (Figure 1B).

**Conclusion:**

Glucose hypometabolism as measured by FDG‐PET is a sensitive neuroimaging marker of the neurodegenerative changes that accompany progressive stages of neurofibrillary tangle pathology in AD. Earliest NFTp‐related neurodegenerative changes captured by FDG‐PET hypometabolism appear to precede macrostructural gray matter atrophy as measured by MRI.